# Phenomenal History, and Post Mortem Measurements, &c., of a Hydrocephalic Subject; with Some Remarks upon the Etiology of Hydrocephalus

**Published:** 1858-10

**Authors:** John T. Banks

**Affiliations:** Zebulon, Ga.


### ARTICLE II.

*Phenomenal history, and Post Mortem Measurements, &c., of a Hydrocephalic Subject; with some remarks upon the Etiology of Hydrocephalus.* By JOHN T. BANKS, M.D., of Zebulon,

The phenomenal history of the following case is obtained from the owner of the negro, and from personal observation, though incomplete. The subject was a negro girl, aged 13. A fine healthy child at birth, and continued so for several weeks, when the attention of her owner was called to the inability of the child to move her lower extremities. The family cannot say that she ever moved her feet: their opinion is that she never did; yet, she nursed well, and grew fast up to the time when her head began to enlarge, which was some time after they noticed that she did not move her feet. The enlargement of her head was rapid during her first and second years; slow, but continuous through the next five; and in the opinion of mother and owner, has not increased any in the last six. During the last five years of her life, I, on several occasions, saw her, and at one time measured the horizontal circumference of her head; but my measurement has been lost. When I first saw her, the sutures and fontanels had completely ossified. Her owner, (an old midwife,) said they had been closed two or three years; but had been very large. If her head increased any in size after I first saw her, it was so little it could not be detected unaided by accurate measurement.

The Hydrocephalic development was not attended by any inflammatory or nervous symptoms noticed by the family. The only thing wrong to them was, that "every thing she ate, went to her head," while her body and limbs seemed to perish. The developement of her body and extremities was scarcely perceptible during the period of rapid encephalic growth; more uniform and comparatively fast after her her head ceased to grow.

She never walked, nor was able to stand alone or hold her head erect. She sat upon the buttock, with her thighs extended forward, inner and posterior part resting on the floor; legs flexed, forming an acute angle with her thighs; feet rotated outwards, so as to rest the inner malleolus and sides of the feet on the floor, but a few inches separated from the outer and posterior part of the thighs. Her body, leaned forward, mostly supported by her upper extremities upon the floor, with her head supported by the occiput resting on her spine. The muscles on the anterior part of the neck, were large and well developed, greatly contrasting with the remainder of her muscular system. Pomum adami, prominent; eyes, natural in appearance, with all of her special senses normal; sleep, quiet, remaining in any position she might be placed in, being unable to get up. She was able to drag herself when placed in the sitting posture, by lifting a part of the weight of her body upon her arms; also, making them serve as points of attachment, to which, she would drag her body. This dragging process caused much thickening of the dependent parts of the skin; which, over the tuber ischii, was at times abraded, but, no doubt produced by the want of cleanliness.

Her organs of *generation* were well developed for one of her size, and equal to a majority of her age, with an unusual amount of pilous growth for girls before puberty.

She talked as well and as intelligibly as could be expected under the circumstances; being surrounded by an unusual amount of ignorance. I could get her to say or do anything she could, by promises of presents of candy, &c., for which she had a great fondness.

Her delight in music, combined with a good memory, formed her most prominent mental characteristic. Any tune she heard once she could sing afterward, and in most instances remember the words.

Her general health was good, without the usual symptoms attending Hydrocephalus, (except the want of power to move her lower limbs,) until a few months before her death, when she had some slight epileptic symptoms; these increased in frequency and no doubt she expired under their immediate effects, not being seen by any of the family at the time.

The post mortem was forty-eight hours after death. The body and extremities relieved from external pressure would contract into their usual position before death; Muscular system pale and poorly developed; Internal organs of generation as well developed as usual for one of her age; Mammary glands in a rudimentary state; Teeth regular, except the two superior canine which projected too much, three of her first molars, carious; Cranial bones thin, not averaging more than a tenth of an inch in thickness where sawed through, marking the largest horizontal circumference; Membranes natural; Brain pale and soft; Weight with membranes 52½ oz avd; the brain formed a thin peripheral case containing several pounds of dropsical flud which bursted, in the effort to remove it, no measurement was made but in the opinion of all present there was 8 to 10 lbs. The convolutions of the brain were unfolded forming a thin wall, less than half an inch in thickness, except the base where the cornua descend into the middle lobes of the brain being about one inch in thickness; The lateral ventricles formed one continuous cavity, the septum being destroyed, containing the dropsical effusion; Cerebellum small compared with the cerebrum; Medulla Oblongata corresponding in size with the cerebellum; Spinal cord not examined. In her vascular system we found one anomaly, the bifurcation of the of the Brachial artery of the left arm within its axillary third.

The cranium clean and dry, measures—

| | | |
|---|---|---|
| Horizontal circumference | 27½ | inches. |
| From one meatus of the ear to the other over vertex | 17½ | " |
| From one meatus of the ear to the other over tuber parietalia | 18½ | " |
| From one meatus of the ear to the other over frontal eminence, | 14 | " |
| Transverse diameter of base, | 4½ | " |

| | | |
|---|---|---|
| From nasal tuberosity to occipital tuberosity under base,.................................. | 8½ | inches. |
| From nasal tuberosity to occipital tuberosity over vertex,....................... ............... | 18½ | " |
| Antero posterior diameter.......................... | 10 | " |
| Transverse through tuber parietalia,............ | 7½ | " |
| Transverse diameter at sagittal suture,........... | 6¾ | " |
| " " across frontal eminence | 2½ | " |
| Length of osfrontis from nasal tuberosity to coronal suture........................................ | 8 | " |
| Mean length of parietal bone 8½ in.; mean width,........................................... | 7 | " |
| From posterior margin of foramen magnum to occipital protuberance,..................... | 3 | " |
| Vertical circumference posterior to foramen magnum,........................ ..................... | 23 | " |
| Circumference of neck over pomum adami,........................ ......................... | 14 | " |

From the above measurements the shape of the head can easily be drawn: Broad posterior, narrow in front, a central vertical division would have two-thirds of its cavity posterior. The face, with its upward look, did not present the usual amount of disproportion common with hydrocephalic subjects; the suture imperfect; serations short and few in coronal and sagittal, with a great many ossa triqueta; bones very thin in places not more than a sixteenth of an inch in thickness. It holds one hundred and forty-three and a half ounces of water.

*What are the causes of Hydroeephalus?* Dr. Bell (Bell & Stokes practice) says, "effusion within the ventricles is the result of tubercles or encephalic tumors in the brain." Cheyne, "that it is peculiarly connected with scrofula." Bennet and Carmicheal deny a necessary connection of Hydrocephalus with inflammation, and suggest tubercolosis and scrofulosis as its most constant cause. Dr. Dickson classes it with the dropsies, and thinks they probably depend "upon some local excitement of the surface affected" and denies to tubercular meningitis the relationship of cause and effect but admits their frequent coincidence. Watson says it is essentially a dropsy. Jones and Seiveking in their pathological anatomy associates it with tubercular meningitis as a frequent exciting cause, and says "that the irritation of tubercular deposits is very liable to induce the secretion of serum either beneath the arachnoid membrane or in the cavities of the brain," and refers to a "malnutrition upon which it is based," evidently meaning its predisposing cause. "Congenital Hydrocephalus is in all probability the result of an inflammation of the meninges during intra-uterine life, or of some malformation difficult to ascertain, bearing some resemblance to a nutritive hypertropy of the encephalon.—(*Billard.*) For the latter hypothesis he reasons ingeniously, at some length ; contending that it "at first consists of an increase of organic energy ; and on the treatment, he says, "How, indeed, can the nutritive activity be suspended in the affected organ, and cause the absorption of the effused fluid ?"

It is not my intention to attempt to reconcile the contradictory theories of the many able and meritorious writers on the Etiology of Hydrocephalus. It may, and often does exist, in subjects where no trace of tuberculosis or scrofulosis can be detected. That it often depends upon tubercular deposits in the meninges we have abundant evidence, and were we without the autopsical evidence, reasoning from analogy, we would be compelled to admit it as one of the many causes productive of Hydrocephalus. The same may be said of encephaloid tumors, or any other pathological condition producing mechanical pressure enough to affect the normal circulation of the brain. An "increase of organic energy" or "nutritive hypertropy" cannot be nearer approximated to Hydrocephalus than to say that it may be a secondary result of a condition previously produced. Hypertrophy in contact with a hard unyielding surface may produce mechanical pressure enough to cause effusion, but that is not the explanation given by Billard ; he says that the "nutritive hypertropy by increasing the power of formation in the encephalic mass will augment the activity of the secretion from the membranes; hence the abundance of fluid at the same time that the organ increases in volume." If exhalation be relatively increased with increase of volume, why is absorption not also? In hypertropy of the brain "the ventricles are found to contain very little or no fluid."—(*Jones & Seiveking's Pathological Anatomy.*) "Hydrocephalus is, in all probability, the result of an inflammation of the meninges during intra uterine life."—(*Billard.*) If so, why should inflammation cease to produce the same result after birth? That inflammation of the meninges often terminates in serous effusion, we have abundant proof in the writings of Craigie, Jones & Seiveking, Watson, and others, and also our own limited experience. The same is also proven by analogy, in inflammation of other serous membranes.

It is my opinion that Hydrocephalus bears the same relationship to *infancy*, that apoplexy does to *adolescency*, resulting from the same exciting cause, *congestion.* The yielding condition of the cranial bones in infancy, and their unyielding condition in mature age, determine the different results from the same cause. The yielding condition of the cranial bones prior to ossific union permit an expansion of their contents, and during congestion an enlargement of the capillary vessels, and consequently an attenuation of their walls favorable to the transudation of the thinner part of their contents; while their unyielding condition in mature age offers a mechanical obstacle to effusion which can only take place at the expense of some other part. As such, a congestion, productive of apoplexy in the adult, might terminate in serous effusion in the infant. This opinion is strengthened by the fact that Hydrocephalus often attacks weak anæmic children; "We are usually told that the subjects of it have been weakly children."—(*Dickson's Elements of Medicine.*) We know that such condition is favorable to local congestion, we know also that the sensorium of children is more susceptible of impressions, and easier excited than that of the adult; and that local congestions are determined by *a*

*priori* sensitiveness of organs; and weak, anæmic children are more subject to Hydrocephalus (other conditions being the same,) than strong and vigorous ones.

The fact that dropsy of the brain has at times presented itsef in the adult, is no argument against the above opinion; for while the effusion is measured by drachms in the adult, it is often measured by pounds in the infant or those cases where the disease originated in infancy; nor can dropsy of the brain exist to an amount beyond a few drachms unless it be *pari passu* with atrophy of the brain or a yielding of its bony case, nor is the doctrine of Special Dyscrasia at variance with the above views, for, if such exist, it is but the predisposing cause.

